# New Players in Neuronal Iron Homeostasis: Insights from CRISPRi Studies

**DOI:** 10.3390/antiox11091807

**Published:** 2022-09-14

**Authors:** Daniel A. Bórquez, Francisco Castro, Marco T. Núñez, Pamela J. Urrutia

**Affiliations:** 1 Center for Biomedical Research, Faculty of Medicine, Universidad Diego Portales, Ejército Libertador 141, Santiago 8370007, Chile; 2 Faculty of Sciences, Universidad de Chile, Las Palmeras 3425, Santiago 7800024, Chile; 3 School of Medical Technology, Faculty of Medicine and Science, Universidad San Sebastián, Lota 2465, Santiago 7510157, Chile

**Keywords:** iron, autophagy, mitochondria, neurons, glycosylphosphatidylinositol, bioinformatics, CRISPR interference

## Abstract

Selective regional iron accumulation is a hallmark of several neurodegenerative diseases, including Alzheimer’s disease and Parkinson’s disease. The underlying mechanisms of neuronal iron dyshomeostasis have been studied, mainly in a gene-by-gene approach. However, recent high-content phenotypic screens using CRISPR/Cas9-based gene perturbations allow for the identification of new pathways that contribute to iron accumulation in neuronal cells. Herein, we perform a bioinformatic analysis of a CRISPR-based screening of lysosomal iron accumulation and the functional genomics of human neurons derived from induced pluripotent stem cells (iPSCs). Consistent with previous studies, we identified mitochondrial electron transport chain dysfunction as one of the main mechanisms triggering iron accumulation, although we substantially expanded the gene set causing this phenomenon, encompassing mitochondrial complexes I to IV, several associated assembly factors, and coenzyme Q biosynthetic enzymes. Similarly, the loss of numerous genes participating through the complete macroautophagic process elicit iron accumulation. As a novelty, we found that the impaired synthesis of glycophosphatidylinositol (GPI) and GPI-anchored protein trafficking also trigger iron accumulation in a cell-autonomous manner. Finally, the loss of critical components of the iron transporters trafficking machinery, including MON2 and PD-associated gene VPS35, also contribute to increased neuronal levels. Our analysis suggests that neuronal iron accumulation can arise from the dysfunction of an expanded, previously uncharacterized array of molecular pathways.

## 1. Introduction

Iron is a micronutrient essential for human health since it participates in several physiological processes, including cellular metabolism, DNA synthesis and repair, neurotransmitter synthesis, and oxygen transportation. Inside of the cell, iron is used as a cofactor for many enzymes given its capacity to participate in electron transfer reactions, switching between two states: ferrous (2+) and ferric (3+) iron. However, this capacity represents a double-edged sword, since it mediates the non-enzymatic conversion of hydrogen peroxide to the highly reactive hydroxyl radical, which is associated with lipid peroxidation, protein oxidation and aggregation, and DNA damage. Therefore, iron content and distribution must be finely regulated to satisfy iron requirements while avoiding iron toxicity.

The cellular iron homeostatic control is driven by two proteins: iron regulatory protein 1 (IRP1) and IRP2. Both are RNA-binding proteins, which, under low-iron-availability conditions bind to conserved stem-loop structures, named iron regulatory elements (IREs), in the untranslated regions (UTRs) of the mRNAs of proteins related to iron homeostasis. Active IRPs increase bioavailable iron levels, by decreasing ferritin levels, a protein responsible for storing intracellular iron, increasing iron incorporation through DMT1 and transferrin receptor (TfR1) and decreasing levels of the iron exporter ferroportin (reviewed in [1]).

The loss of iron homeostasis triggers an increase in redox-active iron and subsequent damage to membrane phospholipids containing polyunsaturated fatty acids, two hallmarks of ferroptosis. The term ferroptosis was first used in 2012 to describe a new type of iron-dependent programmed cell death with characteristics other than necrosis, apoptosis, or autophagy [2]. Since that foundational article, a huge number of studies have described the involvement of ferroptosis in the pathophysiological processes of numerous diseases, including cancer, nervous system diseases, ischemia/reperfusion injury, and many others (reviewed in [3]).

Region-specific iron accumulation in the brain has been demonstrated consistently in neurodegenerative diseases via magnetic resonance imaging (reviewed in [4]), and concordantly an increasing body of literature has outlined the emerging role of ferroptotic cell death in neurodegenerative diseases that have an iron accumulation component [5,6,7]. Moreover, the neuroprotection achieved by the pharmacological or genetic chelation of iron in animal models supports the role of iron in neuronal degeneration [8,9,10,11]. Of particular interest is the novel notion that mitochondrial damage may affect neurodegenerative diseases by regulating many aspects of ferroptosis that include iron dyshomeostasis and lipid peroxidation [12].

A systematic analysis of all of the processes involved in neuronal iron homeostasis is still lacking. Herein, we conducted a bioinformatic analysis integrating data obtained from a genome-wide CRISPR interference (CRISPRi) phenotypic screening of lysosomal iron accumulation in iPSC-derived neurons, with diverse CRISPR-based functional genomics studies to identify new players associated with iron homeostasis maintenance in neurons.

In CRISPRi screens, single-guide RNAs (sgRNAs) target a catalytically dead Cas9 fused to a transcriptional repression domain to transcription start sites in the genome, perturbing gene function by partial knockdown. CRISPRi outperforms the traditional CRISPR knockout approach, which is associated with the activation of DNA damage pathways [13] and have minimal off-target effects typically associated with RNAi technology [14]. The neurons used in the screening were induced from human stem cells by the overexpression of a single transcriptional factor (neurogenin-2) that generated a completely homogenous population of mature excitatory neurons [15].

## 2. Materials and Methods

### 2.1. Data Collection

The CRISPRi screening of iron accumulation (FeRhoNox-1 fluorescence) in induced iPSC-derived glutamatergic neurons was extracted from the CRISPRbrain database (https://crisprbrain.org/) (accessed on 10 January 2022) [16]. This screen included an sgRNA library targeting 730 genes, previously identified as redox or lipid peroxidation modifiers in a principal screen targeting all protein-coding human genes. Positive (increased iron) and negative hits (decreased iron) were manually classified based on the encoded protein function described in Uniprot database [17] release 2022_02. The FeRhoNox-1 probe specifically and quantitatively determined Fe^2+^ levels in the endolysosomes [18]. Differential expressed genes (DEG) associated with CRISPRi of the positive and negative hits were also obtained from CRISPRbrain [16,19]. DEG from SNCA-A53T iPSC-derived dopaminergic neurons were obtained from Fernandes et al. [20]. Redundant siRNA activity (RSA) [21] values of a CRISPR-based screening for the pathway regulators of GFP-NCOA4 abundance in H4 neuroglioma cells were obtained from Goodwin et al. [22]. This screen targeted 18,360 genes, covering most protein-coding genes.

### 2.2. Iron Regulatory Element (IRE) Prediction

mRNA sequences from positive and negative hits of the CRISPRi screening for lysosomal iron accumulation were obtained from GenBank. mRNA sequences were analyzed in the SIRE 2.0 web server (http://ccbg.imppc.org/sires/index.html) (accessed 20 January on 2022) [23] that predicts putative IREs sequences. Only IREs with high overall quality were retained. SIRE program provide 99.3% specificity and 90% precision at a high stringent level.

### 2.3. Pathways Analysis

DEG obtained from CRISPRi coupled to single-cell RNA sequencing were analyzed in the web-based gene set search engine Enrichr (https://maayanlab.cloud/Enrichr/) (accessed 15 April 2022) [24] using the human KEGG pathways 2021. This database includes 8078 genes and 320 pathways. KEGG pathways with a p-adj < 0.05 were considered significantly enriched.

### 2.4. Analysis and Figures

The analysis of correlation between the phenotypic size effect on lysosomal iron levels (Gene Score) and NCOA4 abundance (RSA) from CRISPR-based screenings were performed using GraphPad Prism 9 software. All of the schemes were created with BioRender.com.

## 3. Results and Discussion

### 3.1. Mitochondrial Electron Transport Chain (ETC) Dysfunction Disturbs Neuronal Iron Homeostasis

**Figure 1:**
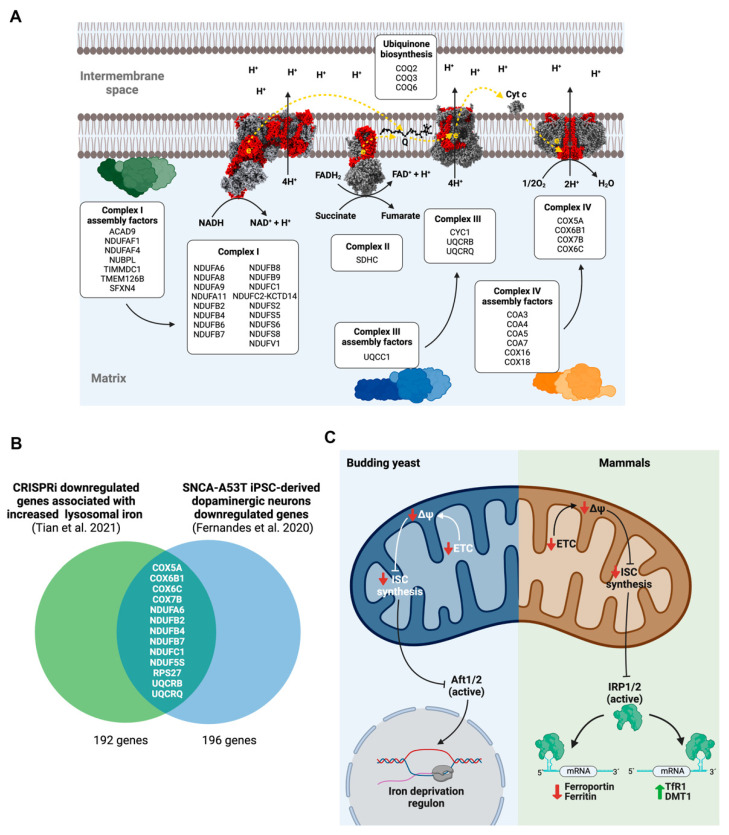
Mitochondrial electron transport chain dysfunction triggers iron accumulation in neurons. (**A**) CRISPRi of several genes associated with mitochondrial electron transport chain components (boxes), including complexes I, II, III and IV subunits (colored red in the atomic structure); numerous assembly factors and enzymes in the ubiquinone biosynthesis pathway induces lysosomal iron accumulation in human iPSC-derived glutamatergic neurons. (**B**) CRISPRi downregulated genes associated with iron accumulation also show diminished expression in a cellular model of PD (SNCA-A53T-expressing dopaminergic neurons). Most of these genes encode mitochondrial ETC subunits [16,20]. (**C**) Comparative model between yeast and human cells of the regulatory mechanism linking dysfunctional mitochondrial ETC with iron regulation through ISCs synthesis.

Mitochondria are the main site of iron utilization, since the synthesis of iron–sulfur clusters (ISC) and heme groups occurs there. Accordingly, cellular iron homeostatic mechanisms, including the IRP/IRE system, are designed to respond to mitochondrial iron deficiency and ensure an adequate supply to this organelle [25,26,27]. Mitochondria also use these iron-bearing cofactors, incorporating into several subunits of the ETC complexes and other mitochondrial enzymes. The limiting nature of iron for ETC function causes the loss of the inner mitochondrial membrane electrochemical potential (ΔΨ, the product of ETC), which triggers an evolutionarily conserved homeostatic mechanism aimed to increase iron bioavailability and uptake. For example, in Saccharomyces cerevisiae, a loss of mitochondrial DNA generates a reduction of the ΔΨ and a transcriptional response of iron starvation mediated by Aft1 and Aft2, two ISC-bearer transcription factors. Both phenomena are connected, since the decrease of ΔΨ would prevent the correct import of proteins involved in iron uptake into mitochondria, the mitochondrial synthesis of ISCs, or ISCs’ export to cytosol, generating an iron-deficiency signal [28]. In mammalian cells, a similar mechanism operates, with IRP1 and IRP2 responding to mitochondrial ISC synthesis as a readout of iron availability [29,30,31] (Figure 1C).

Accordingly, in neurons, CRISPRi of numerous genes that encode subunits encompassing all the mitochondrial ETC complexes, several factors involved in its assembly, and, additionally, ubiquinone biosynthetic enzymes cause iron overload (Figure 1A). Iron accumulation seems to be mediated specifically by ΔΨ loss and not by decreased ATP production, since CRISPRi for genes encoding subunits E and F of ATP synthase do not generate an increase in iron levels (data not shown). Only one gene had previously been associated with iron homeostasis disorders, sideroflexin 4 (SFXN4), which encodes a protein that participates in the assembly of the ND2 module of complex I [32]. SFXN4 knock out (KO) cells show a dramatic redistribution of iron from cytosol to mitochondria [33]. This effect is probably mediated by the c-aconitase-IRP1 switch that has recently been associated with the increased expression of the mitochondrial iron importer, mitoferrin-1 [34].

The connection between ISC synthesis and mitochondrial iron homeostasis is also supported by data from the CRISPRi screening in neurons. Knockdown of GLRX5 and NFU1, two essential components of the mitochondrial ISC protein biogenesis, increases iron levels. The chaperone glutaredoxin 5 (encoded by GLRX5 gene) transiently accommodates [2Fe-2S] cluster, previously assembled de novo on the scaffold protein ISCU2, and subsequently transfers it to client proteins or to late-acting ISC proteins for [4Fe-4S] synthesis. Subsequently, [4Fe-4S] cluster is delivered to complexes I and II of the mitochondrial respiratory chain by NFU1. Previously, it was described that GLRX5-deficient cells exhibit the disruption of iron homeostasis, with a markedly increased IRE-binding activity of IRP1 [35]. Additionally, iron accumulation is a cardinal feature of Friedreich’s ataxia, an autosomal recessive neurodegenerative disease caused by mutations on Frataxin, an allosteric activator of ISCU2 [36].

Mitochondrial dysfunction and iron accumulation contribute to the pathogenesis of additional neurodegenerative diseases, with PD representing the archetypal example. To evaluate the putative contribution of dysregulated gene expression in a PD model to iron accumulation, we analyzed the downregulated genes in iPSC-derived dopaminergic neurons expressing a PD pathogenic ɑ-synuclein mutant (SNCA-A53T), searching for genes associated with increased iron accumulation in the CRISPRi screening. Interestingly, many genes related to mitochondrial ETC functioning are both downregulated in SNCA-A53T dopaminergic neurons and associated with iron accumulation (Figure 1B), potentially linking mitochondrial dysfunction and the iron overload observed in PD [37]. In conclusion, mitochondrial ETC dysfunction and the loss of ΔΨ initiates an iron starvation program that promote iron accumulation through IRP1 (and/or IRP2) activation, as previously observed using complex I inhibitors [38,39].

### 3.2. Autophagy Impairment Results in Lysosomal Iron Overload in Neurons

**Figure 2:**
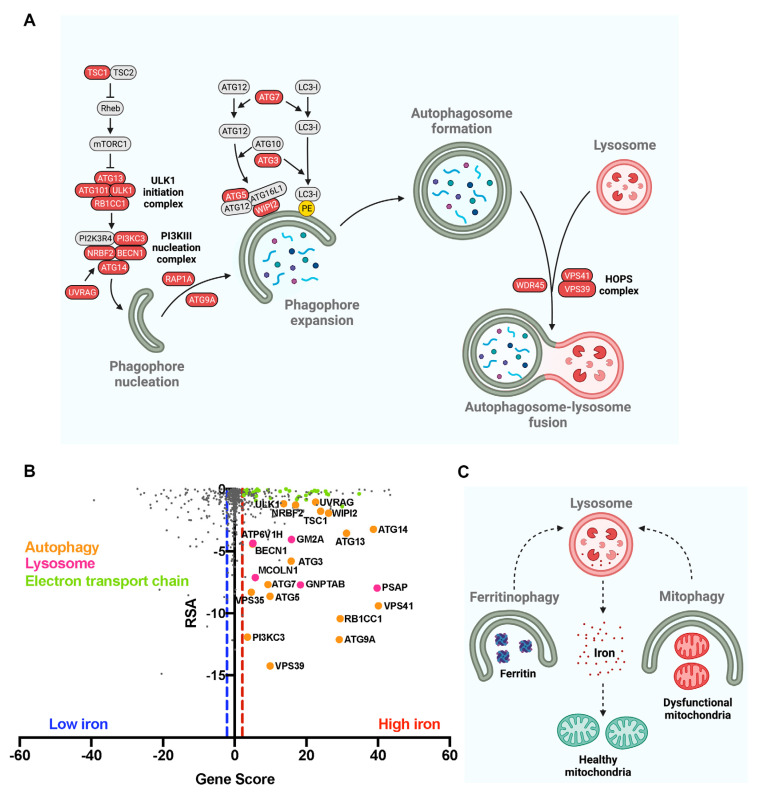
Autophagy impairment results in lysosomal iron overload in neurons. (**A**) CRISPRi downregulation of several genes participating in the macroautophagy process (colored in red) triggers lysosomal iron overload. (**B**) Correlation between phenotypic size effect on lysosomal iron levels (gene score) and NCOA4 abundance (RSA) from CRISPR-based screenings. Red and blue lines separate, respectively, the positive and negative hits from the non-hits of the lysosomal iron screening. (**C**) Two lysosomal degradative pathways, ferritinophagy and mitophagy, connect iron bioavailability with mitochondrial health maintenance.

Our analysis revealed that numerous genes whose repression generates lysosomal iron accumulation participate in the macroautophagic process (Figure 2A).

Several studies have established the intimate relationship between macroautophagy and iron homeostasis. Iron deficiency triggers a PINK1- and Parkin-independent mitochondrial autophagy (mitophagy) preceded by diminished oxygen consumption [40]. Another study also shows that iron chelation induces the selective mitophagy of depolarized mitochondria [41]. Considering that the mitochondria contain significant amounts of iron in the ISCs and heme groups, mitophagy under iron depletion could be a pathway to recycle this metal. Likewise, mitophagy could remove mitochondria with a proteomic immature ETC, since iron-containing cofactors are required for the assembly of all respiratory complexes. Accordingly, defective ISCs assembly activates mitophagy in a IRP1-dependent pathway [42], expanding the iron–mitochondrial relationship to include the lysosomal degradative pathway.

Autophagic turnover of ferritin (ferritinophagy) is also essential for the recycling of cytoplasmic iron stores. Nuclear receptor coactivator 4 (NCOA4) acts as a selective cargo receptor for ferritinophagy [43,44,45] that fine-tunes ferritin degradation. Under iron depletion, NCOA4 is soluble and delivers ferritin to lysosomes via an ATG7-dependent macroautophagy pathway. Under iron repletion, NCOA4 forms insoluble condensates, generated by the binding of iron to its intrinsically disordered region, thus maintaining NCOA4 away from ferritin, allowing its accumulation. Finally, under prolonged iron loading, NCOA4 condensates target ferritin to lysosomes in an ATG7-independent manner [45]. Reinforcing the crosstalk between iron homeostasis and mitochondrial health, NCOA4-mediated ferritin degradation in lysosomes supplies iron to mitochondria under iron-sufficient conditions, allowing the assembly of the mitochondrial respiratory chain complexes, maintaining respiratory activity and membrane potential [46].

In order to evaluate the quantitative contribution of impaired ferritinophagy to iron accumulation, we obtained the RSA values (a probability-based estimation of gene activity) of a genome-wide CRISPR-based screening for the regulators of NCOA4 abundance in H4 cells [22] and paired then with the gene score (integrative measure of size effect and statistical significance) for each gene in the CRISPRi screening of lysosomal iron accumulation (FeRhoNox-1 fluorescence) in iPSC-derived glutamatergic neurons [16]. Interestingly, lysosomal and autophagy-related genes show a striking correlation of their size effect in NCOA4 abundance (lower RSA values) and iron overload (positive gene score) (Figure 2B). Notably, mitochondrial ETC genes are associated with high iron levels but not with NCOA4 abundance, suggesting that mitochondrial dysfunction and ferritinophagy are functionally dissociated.

Our analysis also suggest that some lysosomal proteins can regulate ferritinophagy and iron accumulation in neurons, including N-acetylglucosamine-1-phosphotransferase (encoded by GNPTAB gene), the enzyme that catalyzes the formation of mannose 6-phosphate markers, ganglioside GM2 activator (encoded by GM2A gene), and prosaposin (encoded by PSAP gene), recently associated with lipofuscin and iron accumulation, oxidative stress and ferroptosis in neurons [16].

Furthermore, the CRISPRi screen identified genes previously associated with ferritinophagy and iron accumulation, including WDR45. WDR45 is the causative gene of the most common form of neurodegeneration with brain iron accumulation. Patient-derived fibroblasts present lysosomal Fe^2+^ accumulation, lysosomal enlargement, and a fragmented mitochondrial network [47]. WDR45-deficient neuroblastoma cells show impaired ferritinophagy, increased non-Tf bound iron uptake, elevated mitochondrial iron levels, and deficits in mitochondrial respiration [48]. This phenotype suggests that the inability of cells to obtain the iron stored in ferritin generates a deregulated iron uptake, possibly as a compensatory response. Another mechanism that could contribute to iron accumulation is the deficiency in the autophagic degradation of TfR1, also observed in WDR45-mutant cells [49].

To conclude, iron homeostasis in neurons is highly dependent on the integrity of the macroautophagic system (Figure 2C).

### 3.3. Perturbed Glycosylphosphatidylinositol (GPI) Synthesis and GPI-Anchored Protein Trafficking Increases Neuronal Iron Levels

**Figure 3:**
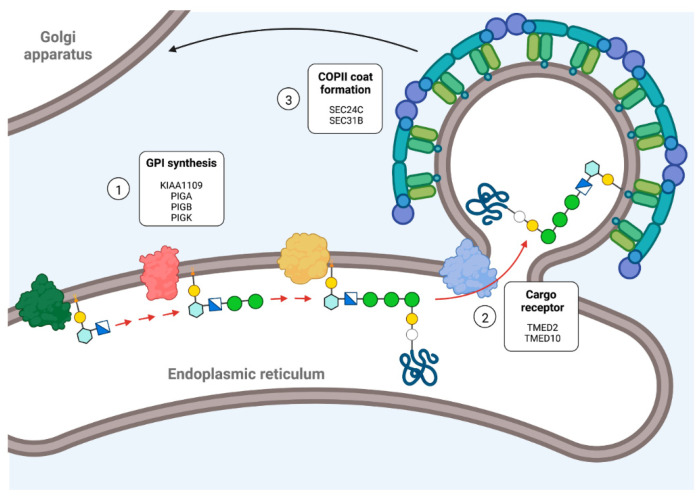
Deficiency in GPI biosynthesis and GPI-anchored protein trafficking causes iron accumulation in neurons. CRISPRi of genes encoding GPI biosynthetic enzymes (1), ER-associated cargo receptors for GPI-anchored proteins (2), and components of the COPII-coated vesicles involved in ER-to-Golgi GPI-anchored protein trafficking (3) results in iron accumulation in neurons.

GPI is a glycolipid membrane anchor synthesized in the endoplasmic reticulum (ER) membrane. Interestingly, CRISPRi of several enzymes involved in GPI synthesis resulted in increased iron levels in iPSC-derived glutamatergic neurons (Figure 3). These included PIGA, the catalytic subunit of the GPI-GlcNAc transferase complex that catalyzes the first of the 11 reactions that generate the GPI; PIGB, which catalyzes the addition of Man3 to Man2; KIAA1109, which facilitates the addition of three ethanolamine phosphate to the nascent GPI [50]; and PIGK, the catalytic subunit of GPI transamidase complex, which finally links the pre-assembled GPI to the target protein.

Newly synthesized GPI-anchored proteins interact with the cargo receptors TMED2 and TMED10 [51] and are selectively packed in COPII-covered vesicles (specifically composed by SEC24C and SEC24D) for ER-to-Golgi trafficking [52]. Interestingly, CRISPRi for TMED2, TMED10, SEC24C, and SEC31B (a structural protein of the COPII cage) also increases iron levels, reinforcing the hypothesis that GPI-anchored proteins have a central role in neuronal iron homeostasis.

The involvement of GPI synthesis in iron homeostasis has been previously documented. Mutations in PIGA resulted in an X-linked syndrome characterized by neurologic dysfunction and systemic iron overload [53,54,55]. This phenotype is attributed to a hepatic deficiency of hemojuvelin (HJV), a GPI-anchored protein [56] that regulates the transcription of hepcidin, a cytokine that acts as the master regulator of systemic iron homeostasis [57]. Accordingly, PIGA KO hepatic cells exhibit diminished hemojuvelin (HJV) expression in cell surface and reduced hepcidin mRNA levels. PIGA KO cells also showed reduced levels of ceruloplasmin, a ferroxidase involved in iron export [54]. Increased serum iron levels were reported in one patient with a mutation in another member of the GPI–GlcNAc transferase complex, PIGH [54]. Iron overload appears to be a hallmark of GPI synthesis deficiency, since patients with mutations in ARV1, a protein involved in flipping GPI-precursors into the ER lumen also shows iron accumulation [58].

In contrast with the proposed systemic mechanism, which ascribes the iron accumulation phenotype to an impaired HJV-hepcidin axis, elevated iron levels observed in iPSC-derived glutamatergic neurons with impaired GPI synthesis or GPI-anchored protein trafficking suggests cell-autonomous mechanisms.

In addition to HJV, many proteins involved in iron homeostasis are GPI-anchored, including an alternative spliced form of ceruloplasmin [59], melanotransferrin [60], intelectin-1 [61], and the cellular prion protein (PrPc) [62,63]. However, GPI-anchored ceruloplasmin expression in the brain is restricted to astrocytes [64,65], and melanotransferrin is mainly present in the brain capillary endothelium [66], excluding a role in neuronal iron homeostasis. Intelectin-1 is the membrane receptor for lactoferrin, another iron binding protein. Interestingly, increased lactoferrin receptor immunoreactivity is observed in the post-mortem mesencephalic tissue of PD patients [67]. Lactoferrin receptor is also increased in the substantia nigra pars compacta of MPTP-treated mice, and lactoferrin has a neuroprotective effect, preventing nigral iron accumulation, dopaminergic neuron loss, and motor impairment in this PD model [68]. Therefore, the lactoferrin-intelectin-1 axis could represent a new neuronal iron homeostatic mechanism, potentially disturbed in GPI synthesis or GPI-anchored protein trafficking dysfunction. On the other hand, PrPc exhibits copper- and NAD(P)H-dependent cell surface and intracellular ferrireductase activity [63] and had been associated with non-Tf-bound iron uptake through DMT1 and ZIP14 in hepatic cells [62]. If this mechanism is also present in neurons, PrPc deficiency could impair iron exit from the endolysosomal system.

### 3.4. Poorly Characterized Proteins That Contribute to Maintain Iron Homeostasis in Neurons

**Figure 4:**
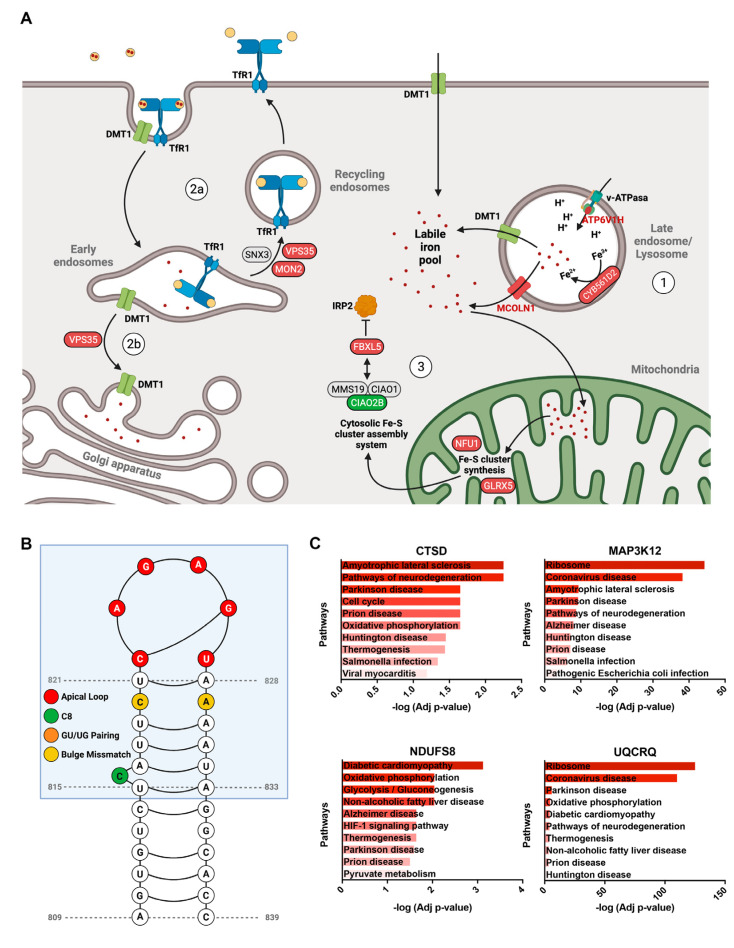
Poorly characterized proteins that contribute to upholding iron homeostasis in neurons. (**A**) Many CRISPRi-targeted genes associated with altered iron levels (increased in red, decreased in green) are connected to the key mechanisms of homeostatic control of this metal. These included lysosomal proteins responsible for iron exit from endolysosomal compartments (1); retromer complex subunits involved in TfR1 (2a) and DMT1 (2b) intracellular trafficking and proteins involved in the regulation of IRP2 levels (3). (**B**) The putative 2D structure of IRE localized in the mRNA of VHL. (**C**) KEGG pathways associated with DEG obtained from CRISPR-mediated genetic perturbations coupled to single-cell RNA sequencing identify a potential link between the genes involved in iron homeostasis and neurodegeneration.

Most proteins involved in neuronal iron homeostasis have been extensively studied; however, our analysis of CRISPRi experiments indicates that previously poorly characterized proteins may also play a relevant role in regulating neuronal iron levels (Figure 4A).

CYB561D2. An essential step in the incorporation of iron into the cytosol, either from transferrin-mediated uptake or through ferritinophagy, consists of the reduction of Fe^3+^ to Fe^2+^ by ferric reductases located in the endolysosomal system. An important class of these enzymes corresponds to a family of transmembrane hemoproteins, called cytochrome b561, which includes DCYTB/CYB561A2 and LCYTB/CYB561A3, known to mediate ferric iron reduction in the apical membrane of enterocytes [69] and the lysosomes of macrophages [70], respectively. Our analysis revealed that the deletion of another cytochrome b561 family member, the transmembrane ferric reductase CYB561D2 [71,72], also induces iron retention into neuronal endolysosomes. Interestingly, CYB561D2 shows no inhibition of its electron transfer reaction under acidic conditions (pH 5.0) in sharp contrast to other cytochrome b561 family members [73], suggesting that it constitutes a special adaptation for functioning in lysosomal acidic environments.

ATP6V1H. The role of acidification in endolysosomal iron transport is reinforced by the phenotype observed in neurons with the reduced expression of ATP6V1H, which encodes an essential subunit of peripheral (V1) ATP-hydrolyzing complex, the vacuolar (v)-ATPase, responsible for endolysosomal acidification. The reduced expression of ATP6V1H triggers iron accumulation into endolysosomal systems in neurons indicating that iron release from these organelles requires acidic pH. Similar results have been observed in Atp6v1h-silenced fibroblasts, where inappropriately acidified endolysosomes generate functional iron deficiency [74].

MCOLN1. After converting lysosomal Fe^3+^ to Fe^2+^, iron is exported to the cytoplasm via DMT1 or MCOLN1. The cation channel MCOLN1 (also known as TRPML1) was initially identified as an iron efflux channel from the endolysosomal system, and MCOLN1-deficient cells shows reduced cytoplasmic Fe^2+^ levels concomitant with endolysosomal iron retention [75]. MCOLN1 KO triggers lysosomal dysfunction, disturbed autophagy, and reduced mitochondrial renewal by mitophagy [76]. CRISPRi of MCOLN1 in neuronal cells replicates the previously observed lysosomal iron retention phenotype.

VPS35. Additionally, to endolysosomal proteins, iron accumulation is observed in neurons with the reduced expression of specific genes associated with the retromer complex, namely VPS35 and MON2, which has been previously associated with the intracellular trafficking of the main iron transporters (TfR1 and DMT1).

The retromer is a protein coat complex essential for endosomal sorting, mediating the endosome-to-trans Golgi network, or endosome-to-plasma membrane recycling. The retromer is assembled by a conserved scaffold of a trimer of vacuolar protein sorting (VPS) proteins VPS35, VPS26, and VPS29 and alternative adaptor modules composed by a sorting nexin (SNX) protein dimer. Interestingly, VPS35 knockdown cells showed a redistribution of labile Fe^2+^ from Golgi to lysosomes, concurrently with an abnormal trafficking of DMT1 toward lysosomes [77].

MON2. MON2 is a peripheral membrane protein, associated with the SNX3-retromer and essential for recycling endosome segregation from the early endosome. In HEK293 MON2 KO cells, TfR1 recycling to the plasma membrane is impaired [78], suggesting its involvement in cell iron homeostasis. SNX3, a binding partner of MON2, also participates in TfR1 recycling and is required for Tf-bound iron uptake [79]. Counterintuitively, CRISPRi of MON2 in iPSC-derived glutamatergic neurons showed increased iron levels.

Surprisingly, no previous studies link iron homeostasis with the CRISPRi genes associated with decreased lysosomal iron levels in neurons, with two notable exceptions: CIAO2B and VHL.

CIAO2B. CIAO2B-depleted HeLa cells showed decreased ferritin levels, increased TfR1 mRNA levels, and increased IRE-binding activity and protein levels of IRP2 [29], a signature of iron deficiency consistent with the phenotype observed in neurons. CIAO2B and its binding partners, MMS19 and CIAO1, bind to FBXL5, the substrate adaptor for a SKP1-CUL1-RBX1 E3 ubiquitin ligase complex that regulates the degradation of IRP2. CIAO2B-MMS19-CIAO1 complex potentiates IRP2 polyubiquitination and degradation [80]; therefore, in CIAO2B-depleted neurons, FBXL5 activity would decrease, increasing IRP2 levels as previously observed. Interestingly, CIAO2B-MMS19-CIAO1 is part of the cytosolic ISC assembly (CIA) targeting complex that delivered ISC to apo-proteins. Recently, a [2Fe-2S] cluster has been identified in the C-terminal, IRP2-recruiting domain of FBXL5 [31], superimposed with the CIAO2B-MMS19-CIAO1 interacting region [80]. Whether this complex is responsible for transferring the [2Fe-2S] cluster to FBXL5 should be the subject of future studies.

VHL. The tumor suppressor gene von Hippel Lindau (VHL) product is a component of the E3 ubiquitin ligase complex that targets the hypoxia-inducible factor (HIF)-1ɑ to proteasomal degradation. Consistent with the phenotype of CRISPRi neurons, hepatocyte-specific VHL-KO shows reduced iron and ferritin levels [81]. Similarly, the loss of VHL in renal carcinoma cells results in increased levels of TfR1, diminished ferritin content, and increased IRE-binding activity of IRP1 and IRP2 [82]. To further search for the putative iron-dependent regulation of genes associated with altered iron levels in neurons, we analyzed the mRNA sequence of positive and negative hits of CRISPRi screen in the search for iron regulatory elements (IREs) through the SIRE web server [23] (Table S1). Interestingly, a high-quality IRE was found in the 3’UTR of VHL mRNA (Figure 4B), suggesting a putative iron-mediated regulation of VHL levels.

CTSD, MAP3K12, NDUFS8, and UQCRQ. Finally, to identify potential mechanisms that connects CRISPR-mediated genetic perturbations to altered iron levels, we analyze single-cell mRNA sequencing data of CRISPRi in iPSC-derived glutamatergic neurons. KEGG pathways associated with the DEGs of four perturbed genes, previously not linked to iron homeostasis, reveals further connections of iron with neurodegenerative diseases (Figure 4C). These include CTSD, which encodes the lysosomal protease cathepsin D; MAP3K12 (also known as Dual leucine zipper protein kinase or DLK); NDUFS8, which encodes a core subunit of the mitochondrial complex I and UQCRQ, a component of the mitochondrial complex III (Figure 4C).

## 4. Conclusions

Systematic analysis of huge data obtained from CRISPR-based screenings in a physiologically relevant system (iPSC-derived human glutamatergic neurons) allowed us to hierarchically organize the processes that impact iron accumulation. Dysfunctional mitochondrial ETC and impaired macroautophagy are the main contributors to iron dyshomeostasis in neurons. However, previously unadvertised mechanisms also contribute to regulating iron levels, namely, GPI-anchored protein metabolism and retromer-dependent iron transporter trafficking. Considering that mitochondrial and lysosomal dysfunction are hallmarks of aging, therapeutic interventions aimed at restoring the homeostasis of these organelles could prevent the iron accumulation (an iron-mediated neurotoxicity) observed in age-associated neurodegenerative diseases. A limitation of our study was that the primary data analyzed derivates from a single neuronal subtype (induced glutamatergic neurons) cultured in vitro. Selective vulnerability of specific neuronal subtypes in neurodegenerative diseases (like dopaminergic or motor neurons) and their differences in the control of iron homeostasis can be addressed using isogenic parental iPSCs differentiated to different neuronal fates.

## Data Availability

The data are contained within the article and supplementary materials.

## Supplementary Materials

The following supporting information can be downloaded at: https://www.mdpi.com/article/10.3390/antiox11091807/s1, Table S1: High confidence iron responsive element (IRE) prediction.

## References

[1] Urrutia P.J., Borquez D.A., Nunez M.T. (2021). Inflaming the Brain with Iron. Antioxidants.

[2] Dixon S.J., Lemberg K.M., Lamprecht M.R., Skouta R., Zaitsev E.M., Gleason C.E., Patel D.N., Bauer A.J., Cantley A.M., Yang W.S. (2012). Ferroptosis: An iron-dependent form of nonapoptotic cell death. Cell.

[3] Li J., Cao F., Yin H.L., Huang Z.J., Lin Z.T., Mao N., Sun B., Wang G. (2020). Ferroptosis: Past, present and future. Cell Death Dis..

[4] Moller H.E., Bossoni L., Connor J.R., Crichton R.R., Does M.D., Ward R.J., Zecca L., Zucca F.A., Ronen I. (2019). Iron, Myelin, and the Brain: Neuroimaging Meets Neurobiology. Trends Neurosci..

[5] Ward R.J., Zucca F.A., Duyn J.H., Crichton R.R., Zecca L. (2014). The role of iron in brain ageing and neurodegenerative disorders. Lancet Neurol..

[6] Wang Y., Tang B., Zhu J., Yu J., Hui J., Xia S., Ji J. (2022). Emerging Mechanisms and Targeted Therapy of Ferroptosis in Neurological Diseases and Neuro-oncology. Int. J. Biol. Sci..

[7] Vitalakumar D., Sharma A., Flora S.J.S. (2021). Ferroptosis: A potential therapeutic target for neurodegenerative diseases. J. Biochem. Mol. Toxicol..

[8] Chen J., Marks E., Lai B., Zhang Z., Duce J.A., Lam L.Q., Volitakis I., Bush A.I., Hersch S., Fox J.H. (2013). Iron accumulates in Huntington’s disease neurons: Protection by deferoxamine. PLoS ONE.

[9] Mechlovich D., Amit T., Bar-Am O., Mandel S., Youdim M.B., Weinreb O. (2014). The novel multi-target iron chelator, M30 modulates HIF-1alpha-related glycolytic genes and insulin signaling pathway in the frontal cortex of APP/PS1 Alzheimer’s disease mice. Curr. Alzheimer Res..

[10] Kaur D., Yantiri F., Rajagopalan S., Kumar J., Mo J.Q., Boonplueang R., Viswanath V., Jacobs R., Yang L., Beal M.F. (2003). Genetic or pharmacological iron chelation prevents MPTP-induced neurotoxicity in vivo: A novel therapy for Parkinson’s disease. Neuron.

[11] Devos D., Moreau C., Devedjian J.C., Kluza J., Petrault M., Laloux C., Jonneaux A., Ryckewaert G., Garcon G., Rouaix N. (2014). Targeting chelatable iron as a therapeutic modality in Parkinson’s disease. Antioxid. Redox Signal..

[12] Zhou J., Jin Y., Lei Y., Liu T., Wan Z., Meng H., Wang H. (2020). Ferroptosis Is Regulated by Mitochondria in Neurodegenerative Diseases. Neurodegener. Dis..

[13] Haapaniemi E., Botla S., Persson J., Schmierer B., Taipale J. (2018). CRISPR-Cas9 genome editing induces a p53-mediated DNA damage response. Nat. Med..

[14] Gilbert L.A., Horlbeck M.A., Adamson B., Villalta J.E., Chen Y., Whitehead E.H., Guimaraes C., Panning B., Ploegh H.L., Bassik M.C. (2014). Genome-Scale CRISPR-Mediated Control of Gene Repression and Activation. Cell.

[15] Zhang Y., Pak C., Han Y., Ahlenius H., Zhang Z., Chanda S., Marro S., Patzke C., Acuna C., Covy J. (2013). Rapid single-step induction of functional neurons from human pluripotent stem cells. Neuron.

[16] Tian R., Abarientos A., Hong J., Hashemi S.H., Yan R., Drager N., Leng K., Nalls M.A., Singleton A.B., Xu K. (2021). Genome-wide CRISPRi/a screens in human neurons link lysosomal failure to ferroptosis. Nat. Neurosci..

[17] The UniProt Consortium (2021). UniProt: The universal protein knowledgebase in 2021. Nucleic Acids Res..

[18] Halcrow P.W., Kumar N., Afghah Z., Fischer J.P., Khan N., Chen X., Meucci O., Geiger J.D. (2022). Heterogeneity of ferrous iron-containing endolysosomes and effects of endolysosome iron on endolysosome numbers, sizes, and localization patterns. J. Neurochem..

[19] Tian R., Gachechiladze M.A., Ludwig C.H., Laurie M.T., Hong J.Y., Nathaniel D., Prabhu A.V., Fernandopulle M.S., Patel R., Abshari M. (2019). CRISPR Interference-Based Platform for Multimodal Genetic Screens in Human iPSC-Derived Neurons. Neuron.

[20] Fernandes H.J.R., Patikas N., Foskolou S., Field S.F., Park J.-E., Byrne M.L., Bassett A.R., Metzakopian E. (2020). Single-Cell Transcriptomics of Parkinson’s Disease Human In Vitro Models Reveals Dopamine Neuron-Specific Stress Responses. Cell Rep..

[21] Konig R., Chiang C.-Y., Tu B.P., Yan S.F., DeJesus P.D., Romero A., Bergauer T., Orth A., Krueger U., Zhou Y. (2007). A probability-based approach for the analysis of large-scale RNAi screens. Nat. Methods.

[22] Goodwin J.M., Dowdle W.E., DeJesus R., Wang Z., Bergman P., Kobylarz M., Lindeman A., Xavier R.J., McAllister G., Nyfeler B. (2017). Autophagy-Independent Lysosomal Targeting Regulated by ULK1/2-FIP200 and ATG9. Cell Rep..

[23] Campillos M., Cases I., Hentze M.W., Sanchez M. (2010). SIREs: Searching for iron-responsive elements. Nucleic Acids Res..

[24] Xie Z., Bailey A., Kuleshov M.V., Clarke D.J.B., Evangelista J.E., Jenkins S.L., Lachmann A., Wojciechowicz M.L., Kropiwnicki E., Jagodnik K.M. (2021). Gene Set Knowledge Discovery with Enrichr. Curr. Protoc..

[25] Li H., Zhao H., Hao S., Shang L., Wu J., Song C., Meyron-Holtz E.G., Qiao T., Li K. (2018). Iron regulatory protein deficiency compromises mitochondrial function in murine embryonic fibroblasts. Sci. Rep..

[26] Martelli A., Schmucker S., Reutenauer L., Mathieu J.R.R., Peyssonnaux C., Karim Z., Puy H., Galy B., Hentze M.W., Puccio H. (2015). Iron regulatory protein 1 sustains mitochondrial iron loading and function in frataxin deficiency. Cell Metab..

[27] Galy B., Ferring-Appel D., Sauer S.W., Kaden S., Lyoumi S., Puy H., Kolker S., Grone H.J., Hentze M.W. (2010). Iron regulatory proteins secure mitochondrial iron sufficiency and function. Cell Metab..

[28] Veatch J.R., McMurray M.A., Nelson Z.W., Gottschling D.E. (2009). Mitochondrial dysfunction leads to nuclear genome instability via an iron-sulfur cluster defect. Cell.

[29] Stehling O., Mascarenhas J., Vashisht A.A., Sheftel A.D., Niggemeyer B., Rosser R., Pierik A.J., Wohlschlegel J.A., Lill R. (2013). Human CIA2A-FAM96A and CIA2B-FAM96B integrate iron homeostasis and maturation of different subsets of cytosolic-nuclear iron-sulfur proteins. Cell Metab..

[30] Terzi E.M., Sviderskiy V.O., Alvarez S.W., Whiten G.C., Possemato R. (2021). Iron-sulfur cluster deficiency can be sensed by IRP2 and regulates iron homeostasis and sensitivity to ferroptosis independent of IRP1 and FBXL5. Sci. Adv..

[31] Wang H., Shi H., Rajan M., Canarie E.R., Hong S., Simoneschi D., Pagano M., Bush M.F., Stoll S., Leibold E.A. (2020). FBXL5 Regulates IRP2 Stability in Iron Homeostasis via an Oxygen-Responsive [2Fe2S] Cluster. Mol. Cell.

[32] Jackson T.D., Crameri J.J., Muellner-Wong L., Frazier A.E., Palmer C.S., Formosa L.E., Hock D.H., Fujihara K.M., Stait T., Sharpe A.J. (2022). Sideroflexin 4 is a complex I assembly factor that interacts with the MCIA complex and is required for the assembly of the ND2 module. Proc. Natl. Acad. Sci. USA.

[33] Paul B.T., Tesfay L., Winkler C.R., Torti F.M., Torti S.V. (2019). Sideroflexin 4 affects Fe-S cluster biogenesis, iron metabolism, mitochondrial respiration and heme biosynthetic enzymes. Sci. Rep..

[34] Zhang T., Sun L., Hao Y., Suo C., Shen S., Wei H., Ma W., Zhang P., Wang T., Gu X. (2022). ENO1 suppresses cancer cell ferroptosis by degrading the mRNA of iron regulatory protein 1. Nat. Cancer.

[35] Ye H., Jeong S.Y., Ghosh M.C., Kovtunovych G., Silvestri L., Ortillo D., Uchida N., Tisdale J., Camaschella C., Rouault T.A. (2010). Glutaredoxin 5 deficiency causes sideroblastic anemia by specifically impairing heme biosynthesis and depleting cytosolic iron in human erythroblasts. J. Clin. Investig..

[36] Fox N.G., Yu X., Feng X., Bailey H.J., Martelli A., Nabhan J.F., Strain-Damerell C., Bulawa C., Yue W.W., Han S. (2019). Structure of the human frataxin-bound iron-sulfur cluster assembly complex provides insight into its activation mechanism. Nat. Commun..

[37] Bi M., Du X., Jiao Q., Liu Z., Jiang H. (2020). alpha-Synuclein Regulates Iron Homeostasis via Preventing Parkin-Mediated DMT1 Ubiquitylation in Parkinson’s Disease Models. ACS Chem. Neurosci..

[38] Urrutia P.J., Aguirre P., Tapia V., Carrasco C.M., Mena N.P., Nunez M.T. (2017). Cell death induced by mitochondrial complex I inhibition is mediated by Iron Regulatory Protein 1. Biochim. Biophys. Acta Mol. Basis Dis..

[39] Mena N.P., Bulteau A.L., Salazar J., Hirsch E.C., Nunez M.T. (2011). Effect of mitochondrial complex I inhibition on Fe-S cluster protein activity. Biochem. Biophys. Res. Commun..

[40] Allen G.F.G., Toth R., James J., Ganley I.G. (2013). Loss of iron triggers PINK1/Parkin-independent mitophagy. EMBO Rep..

[41] Hara Y., Yanatori I., Tanaka A., Kishi F., Lemasters J.J., Nishina S., Sasaki K., Hino K. (2020). Iron loss triggers mitophagy through induction of mitochondrial ferritin. EMBO Rep..

[42] Wu H., Wei H., Zhang D., Sehgal S.A., Zhang D., Wang X., Qin Y., Liu L., Chen Q. (2020). Defective mitochondrial ISCs biogenesis switches on IRP1 to fine tune selective mitophagy. Redox Biol..

[43] Mancias J.D., Wang X., Gygi S.P., Harper J.W., Kimmelman A.C. (2014). Quantitative proteomics identifies NCOA4 as the cargo receptor mediating ferritinophagy. Nature.

[44] Dowdle W.E., Nyfeler B., Nagel J., Elling R.A., Liu S., Triantafellow E., Menon S., Wang Z., Honda A., Pardee G. (2014). Selective VPS34 inhibitor blocks autophagy and uncovers a role for NCOA4 in ferritin degradation and iron homeostasis in vivo. Nat. Cell Biol..

[45] Kuno S., Fujita H., Tanaka Y.K., Ogra Y., Iwai K. (2022). Iron-induced NCOA4 condensation regulates ferritin fate and iron homeostasis. EMBO Rep..

[46] Fujimaki M., Furuya N., Saiki S., Amo T., Imamichi Y., Hattori N. (2019). Iron Supply via NCOA4-Mediated Ferritin Degradation Maintains Mitochondrial Functions. Mol. Cell. Biol..

[47] Lee H.E., Jung M.K., Noh S.G., Choi H.B., Chae S.H., Lee J.H., Mun J.Y. (2021). Iron Accumulation and Changes in Cellular Organelles in WDR45 Mutant Fibroblasts. Int. J. Mol. Sci..

[48] Aring L., Choi E.K., Kopera H., Lanigan T., Iwase S., Klionsky D.J., Seo Y.A. (2022). A neurodegeneration gene, WDR45, links impaired ferritinophagy to iron accumulation. J. Neurochem..

[49] Xiong Q., Li X., Li W., Chen G., Xiao H., Li P., Wu C. (2021). WDR45 Mutation Impairs the Autophagic Degradation of Transferrin Receptor and Promotes Ferroptosis. Front. Mol. Biosci..

[50] Toulmay A., Whittle F.B., Yang J., Bai X., Diarra J., Banerjee S., Levine T.P., Golden A., Prinz W.A. (2022). Vps13-like proteins provide phosphatidylethanolamine for GPI anchor synthesis in the ER. J. Cell Biol..

[51] Nagae M., Hirata T., Morita-Matsumoto K., Theiler R., Fujita M., Kinoshita T., Yamaguchi Y. (2016). 3D Structure and Interaction of p24beta and p24delta Golgi Dynamics Domains: Implication for p24 Complex Formation and Cargo Transport. J. Mol. Biol..

[52] Bonnon C., Wendeler M.W., Paccaud J.-P., Hauri H.-P. (2010). Selective export of human GPI-anchored proteins from the endoplasmic reticulum. J. Cell Sci..

[53] Swoboda K.J., Margraf R.L., Carey J.C., Zhou H., Newcomb T.M., Coonrod E., Durtschi J., Mallempati K., Kumanovics A., Katz B.E. (2014). A novel germline PIGA mutation in Ferro-Cerebro-Cutaneous syndrome: A neurodegenerative X-linked epileptic encephalopathy with systemic iron-overload. Am. J. Med. Genet. A.

[54] Muckenthaler L., Marques O., Colucci S., Kunz J., Fabrowski P., Bast T., Altamura S., Hochsmann B., Schrezenmeier H., Langlotz M. (2022). Constitutional PIGA mutations cause a novel subtype of hemochromatosis in patients with neurologic dysfunction. Blood.

[55] Flores-Torres J., Carver J.D., Sanchez-Valle A. (2021). PIGA Mutations Can Mimic Neonatal Hemochromatosis. Pediatrics.

[56] Silvestri L., Pagani A., Fazi C., Gerardi G., Levi S., Arosio P., Camaschella C. (2007). Defective targeting of hemojuvelin to plasma membrane is a common pathogenetic mechanism in juvenile hemochromatosis. Blood.

[57] Babitt J.L., Huang F.W., Wrighting D.M., Xia Y., Sidis Y., Samad T.A., Campagna J.A., Chung R.T., Schneyer A.L., Woolf C.J. (2006). Bone morphogenetic protein signaling by hemojuvelin regulates hepcidin expression. Nat. Genet..

[58] Davids M., Menezes M., Guo Y., McLean S.D., Hakonarson H., Collins F., Worgan L., Billington C.J., Maric I., Littlejohn R.O. (2020). Homozygous splice-variants in human ARV1 cause GPI-anchor synthesis deficiency. Mol. Genet. Metab..

[59] Jeong S.Y., David S. (2003). Glycosylphosphatidylinositol-anchored ceruloplasmin is required for iron efflux from cells in the central nervous system. J. Biol. Chem..

[60] Kennard M.L., Richardson D.R., Gabathuler R., Ponka P., Jefferies W.A. (1995). A novel iron uptake mechanism mediated by GPI-anchored human p97. EMBO J..

[61] Akiyama Y., Oshima K., Kuhara T., Shin K., Abe F., Iwatsuki K., Nadano D., Matsuda T. (2013). A lactoferrin-receptor, intelectin 1, affects uptake, sub-cellular localization and release of immunochemically detectable lactoferrin by intestinal epithelial Caco-2 cells. J. Biochem..

[62] Tripathi A.K., Haldar S., Qian J., Beserra A., Suda S., Singh A., Hopfer U., Chen S.G., Garrick M.D., Turner J.R. (2015). Prion protein functions as a ferrireductase partner for ZIP14 and DMT1. Free Radic. Biol. Med..

[63] Singh A., Haldar S., Horback K., Tom C., Zhou L., Meyerson H., Singh N. (2013). Prion protein regulates iron transport by functioning as a ferrireductase. J. Alzheimers Dis..

[64] Bennett M.V., Sandri C. (1989). The electromotor system of the electric eel investigated with horseradish peroxidase as a retrograde tracer. Brain Res..

[65] Patel B.N., David S. (1997). A novel glycosylphosphatidylinositol-anchored form of ceruloplasmin is expressed by mammalian astrocytes. J. Biol. Chem..

[66] Rothenberger S., Food M.R., Gabathuler R., Kennard M.L., Yamada T., Yasuhara O., McGeer P.L., Jefferies W.A. (1996). Coincident expression and distribution of melanotransferrin and transferrin receptor in human brain capillary endothelium. Brain Res..

[67] Faucheux B.A., Nillesse N., Damier P., Spik G., Mouatt-Prigent A., Pierce A., Leveugle B., Kubis N., Hauw J.J., Agid Y. (1995). Expression of lactoferrin receptors is increased in the mesencephalon of patients with Parkinson disease. Proc. Natl. Acad. Sci. USA.

[68] Liu H., Wu H., Zhu N., Xu Z., Wang Y., Qu Y., Wang J. (2020). Lactoferrin protects against iron dysregulation, oxidative stress, and apoptosis in 1-methyl-4-phenyl-1,2,3,6-tetrahydropyridine (MPTP)-induced Parkinson’s disease in mice. J. Neurochem..

[69] McKie A.T., Barrow D., Latunde-Dada G.O., Rolfs A., Sager G., Mudaly E., Mudaly M., Richardson C., Barlow D., Bomford A. (2001). An iron-regulated ferric reductase associated with the absorption of dietary iron. Science.

[70] Meng F., Fleming B.A., Jia X., Rousek A.A., Mulvey M.A., Ward D.M. (2022). Lysosomal iron recycling in mouse macrophages is dependent upon both LcytB and Steap3 reductases. Blood Adv..

[71] Mizutani A., Sanuki R., Kakimoto K., Kojo S., Taketani S. (2007). Involvement of 101F6, a homologue of cytochrome b561, in the reduction of ferric ions. J. Biochem..

[72] El Behery M., Fujimura M., Kimura T., Tsubaki M. (2020). Direct measurements of ferric reductase activity of human 101F6 and its enhancement upon reconstitution into phospholipid bilayer nanodisc. Biochem. Biophys. Rep..

[73] Recuenco M.C., Rahman M.M., Takeuchi F., Kobayashi K., Tsubaki M. (2013). Electron transfer reactions of candidate tumor suppressor 101F6 protein, a cytochrome b561 homologue, with ascorbate and monodehydroascorbate radical. Biochemistry.

[74] Yambire K.F., Rostosky C., Watanabe T., Pacheu-Grau D., Torres-Odio S., Sanchez-Guerrero A., Senderovich O., Meyron-Holtz E.G., Milosevic I., Frahm J. (2019). Impaired lysosomal acidification triggers iron deficiency and inflammation in vivo. eLife.

[75] Dong X.-P., Cheng X., Mills E., Delling M., Wang F., Kurz T., Xu H. (2008). The type IV mucolipidosis-associated protein TRPML1 is an endolysosomal iron release channel. Nature.

[76] Siow W.X., Kabiri Y., Tang R., Chao Y.-K., Plesch E., Eberhagen C., Flenkenthaler F., Frohlich T., Bracher F., Grimm C. (2022). Lysosomal TRPML1 regulates mitochondrial function in hepatocellular carcinoma cells. J. Cell Sci..

[77] Hirayama T., Inden M., Tsuboi H., Niwa M., Uchida Y., Naka Y., Hozumi I., Nagasawa H. (2019). A Golgi-targeting fluorescent probe for labile Fe(ii) to reveal an abnormal cellular iron distribution induced by dysfunction of VPS35. Chem. Sci..

[78] Zhao S.-B., Dean N., Gao X.-D., Fujita M. (2020). MON2 Guides Wntless Transport to the Golgi through Recycling Endosomes. Cell Struct. Funct..

[79] Chen C., Garcia-Santos D., Ishikawa Y., Seguin A., Li L., Fegan K.H., Hildick-Smith G.J., Shah D.I., Cooney J.D., Chen W. (2013). Snx3 regulates recycling of the transferrin receptor and iron assimilation. Cell Metab..

[80] Mayank A.K., Pandey V., Vashisht A.A., Barshop W.D., Rayatpisheh S., Sharma T., Haque T., Powers D.N., Wohlschlegel J.A. (2019). An Oxygen-Dependent Interaction between FBXL5 and the CIA-Targeting Complex Regulates Iron Homeostasis. Mol. Cell.

[81] Peyssonnaux C., Zinkernagel A.S., Schuepbach R.A., Rankin E., Vaulont S., Haase V.H., Nizet V., Johnson R.S. (2007). Regulation of iron homeostasis by the hypoxia-inducible transcription factors (HIFs). J. Clin. Investig..

[82] Alberghini A., Recalcati S., Tacchini L., Santambrogio P., Campanella A., Cairo G. (2005). Loss of the von Hippel Lindau tumor suppressor disrupts iron homeostasis in renal carcinoma cells. J. Biol. Chem..

