# Supplementary material for: New Players in Neuronal Iron Homeostasis: Insights from CRISPRi Studies
Source: Antioxidants (Basel). 2022 Sep 14;11(9):1807. doi: 10.3390/antiox11091807 (PMC9495848; doi:10.3390/antiox11091807)
Supplement: Supplementary file 1 [file antioxidants-11-01807-s001.zip › antioxidants-1878272-supplementary.pdf]

Supplementary Table S1. High confidence iron responsive element (IRE) prediction

Approved symbol	HGNC ID	Sequence	Position in seq.	CDS	Relative position to CDS	Hit Class
ADSL	HGNC:291	TCTCAGCCTCCCAAAGTTCTGGGATTATAGA	2377 - 2408	60 - 1514	3' UTR	Negative
ALG9	HGNC:15672	CAACACACCTGCTCAGAGTAGCAGTTCTCCC	4712-4743	100 - 1,935	3' UTR	Positive
CEP295	HGNC:29366	AGCCTTACAGATCCAGAGTCATTTTCAGAGC	6051 - 6082	120 - 7925	Inside CDS	Negative
CYB5RL	HGNC:32220	CCCAGTGCTGCGGCAGTGGCTGCTCACCCTG	354-385	278 - 1225	Inside CDS	Positive
FOXK1	HGNC:23480	CACCAGTCGGGCCGAGTGTGGCCTCATGGTT	5964-5995	15 - 2216	3' UTR	Positive
GPAT4	HGNC:20880	CCTTGGCCTCCCAAAGTTCTGGGATTACAGG	5382-5413	928 - 2298	3' UTR	Positive
KLF7	HGNC:6350	TCCATCACCTCTGCAGTGGCAGAGATGGTTA	4395 - 4426	381 - 1289	3' UTR	Negative
MFHAS1	HGNC:16982	ATCGGGTCGGGGCGAGAGTGCCCCACGCGGT	2134-2165	198 - 3644	Inside CDS	Positive
MRGBP	HGNC:15866	TTGATACCAAAGTAAGTTCACTTTTCCTCCA	1634-1665	52 - 666	3' UTR	Positive
MYO1C	HGNC:7597	CAATCGGCCCTGGGAGCCACCAGGTGGGAAA	4599-4630	200 - 3391	3' UTR	Positive
RFX7	HGNC:25777	TCTTTCTCTTGGACAGTGATTCAAAGTCAGT	2728-2759	573 - 3731	Inside CDS	Positive
VHL	HGNC:12687	AGTGTCTCATTCTCAGAGTAAAATAGGCACC	809 - 840	71 - 712	3' UTR	Negative
